# Investigation of potential sex-based differences in trastuzumab-induced chronic cardiotoxicity in a rat model

**DOI:** 10.3389/fphar.2026.1809964

**Published:** 2026-06-26

**Authors:** Réka Losonczi, Zsolt Galla, Merse Kis, Klaudia Kupecz, Dávid Volford, Andrea Siska, Réka Somogyi, Imre Földesi, Gábor Cserni, András Kriston, Ferenc Kovács, Péter Horváth, Péter Monostori, Zsuzsanna Kahán, Márta Sárközy

**Affiliations:** 1 Cardiovascular Research Group, Department of Pathophysiology, Albert Szent-Györgyi Medical School, University of Szeged, Szeged, Hungary; 2 Department of Biochemistry, Albert Szent-Györgyi Medical School, University of Szeged, Szeged, Hungary; 3 Metabolic and Newborn Screening Laboratory, Department of Pediatrics, Albert Szent-Györgyi Medical School, University of Szeged, Szeged, Hungary; 4 Department of Laboratory Medicine, Albert Szent-Györgyi Medical School, University of Szeged, Szeged, Hungary; 5 Department of Pathology, Albert Szent-Györgyi Medical School, University of Szeged, Szeged, Hungary; 6 Synthetic and Systems Biology Unit, HUN-REN Biological Research Centre, Szeged, Hungary; 7 Single-Cell Technologies Ltd., Szeged, Hungary; 8 Institute for Molecular Medicine Finland, University of Helsinki, Helsinki, Finland; 9 Institute of AI for Health, Helmholtz Zentrum München, Neuherberg, Germany; 10 Department of Oncotherapy, Albert Szent-Györgyi Medical School, University of Szeged, Szeged, Hungary

**Keywords:** glucose and fatty acid metabolism, heart failure, kynurenine pathway, trastuzumab, trastuzumab-induced chronic cardiotoxicity, tryptophan and arginine metabolites

## Abstract

**Background:**

Although trastuzumab (TZB) significantly increases survival in patients with human epidermal growth factor receptor 2 (HER2/ErbB2)-positive breast and gastrointestinal cancers, its use may be limited by chronic cardiotoxicity. Several tryptophan (Trp) metabolites are associated with oxidative stress, inflammation, and metabolic disturbances in heart failure (HF). Here, we aimed to characterize changes in left ventricular (LV) concentrations of selected Trp metabolites in a rat model of TZB-induced chronic cardiotoxicity.

**Methods:**

Male and female Wistar-Hannover rats (300–400 g) were divided into 2-2 groups: i) physiological saline-treated (6 × 1 mL/kg, i.p.) control, and ii) TZB-treated (2 mg/kg, then 5 × 1 mg/kg, i.p.) groups. At weeks 12 and 19, echocardiography was performed. At week 20, blood and LV samples were collected. Then, histology, RT-qPCR, and UHPLC-MS/MS analyses of genes and metabolites related to nitro-oxidative stress, inflammation, glucose and fatty acid metabolism, and Trp metabolism were performed.

**Results:**

Diastolic dysfunction began in both TZB-treated groups by week 12. At the endpoint, both TZB-treated groups showed echocardiographic, histologic, and molecular signs of chronic cardiotoxicity, accompanied by LV overexpression of genes associated with inflammation and nitro-oxidative stress, repression of glucose transporter 4, glycerol-3-phosphate dehydrogenase, and carnitine palmitoyltransferase. However, only female TZB-treated rats showed increased LV levels of Trp, 3-hydroxykynurenine, quinolinic acid, and nicotinamide adenine dinucleotide (NAD^+^). In contrast, TZB-treated males presented lower LV levels of kynurenine and xanthurenic acid.

**Conclusion:**

Comparable TZB-induced chronic cardiotoxicity developed in both sexes. However, LV Trp metabolite concentrations showed sex-divergent alterations, the significance of which should be clarified in further mechanistic studies.

## 1 Introduction

Worldwide, approximately 20 million patients were newly diagnosed with malignant tumors, and close to 10 million died from cancer in 2022. Most frequently, lung cancer was diagnosed, followed by breast cancer and colorectal tumors (Bray et al., 2024). While modern oncotherapy may lead to better cancer survival, long-term complications, including cardiovascular diseases (CVDs), often appear after cancer treatment and might result in the premature death of the patient (Florido et al., 2022; Aleman et al., 2014).

The human epidermal growth factor receptor (HER/ErbB) family, comprising HER1, HER2, HER3, and HER4, is widely expressed throughout the body and regulates processes such as metabolism, cell growth, division, differentiation, and apoptosis (Slavcheva and Angelov, 2023). Overexpression of the HER2 protein or amplification of its gene occurs in approximately one-quarter of primary breast carcinomas. It is associated with reduced disease-free and overall survival if not treated with modern anti-HER2 therapies (Escrivá-de-Romaní et al., 2018; Vici et al., 2015; Yan et al., 2015). HER2 positivity has also been observed in other cancer types, including gastrointestinal, ovarian, endometrial, bladder, lung, colon, and head and neck tumors (Yan et al., 2015; Zhao et al., 2019). Recently, even low levels of HER2 protein expression can be used to target cancer cells with antibody-drug conjugates (Tarantino et al., 2022).

TZB is a recombinant humanized IgG1 monoclonal antibody against HER2, used to treat various HER2-positive cancers (Early Breast Cancer Trialists’ Collab orative group EBCTCG, 2021; Ba and ng, 2012). It is used not only in combinations with cytotoxic agents but also in dual HER2 blockade (with pertuzumab or, rarely, with the tyrosine kinase inhibitor lapatinib), and as a target for receptor-mediated endocytosis of TZB-linked cytotoxic agents in antibody-drug conjugates (Tarantino et al., 2022). TZB reduces HER2 expression in the cell membrane and promotes antibody-dependent cell-mediated phagocytosis and cytotoxicity (Slavcheva and Angelov, 2023; Escrivá-de-Romaní et al., 2018). TZB also reduces vascular endothelial growth factor (VEGF) production, inhibits HER2-mediated cell survival (e.g., via the PI3K/AKT pathway) and adenosine monophosphate-activated protein kinase (AMPK) signaling, and reduces ATP synthesis by repressing glycolysis and β-oxidation in tumor cells (Slavcheva and Angelov, 2023; Choksey and Timm, 2021).

In the cardiovascular system, HER2-expressing cell types include cardiomyocytes (CMs), endothelial cells, and cardiac progenitor cells (Lemmens et al., 2007). Neuregulin-1 (NRG-1) is a transmembrane protein secreted by endothelial cells (Lemmens et al., 2007). NRG-1 shows high affinity for HER3 in tumor cells and for HER4 in CMs, and it promotes heterodimer formation (Slavcheva and Angelov, 2023; Lemmens et al., 2007; Berdiel-Acer et al., 2021). In the cardiovascular system, by blocking HER2-HER4 heterodimerization, TZB may inhibit antiapoptotic survival pathways, disrupt energy homeostasis, activate immune cells, induce inflammation via reactive oxygen species generation, and stimulate fibroblasts, thereby promoting collagen deposition in the extracellular matrix (Slavcheva and Angelov, 2023; Lemmens et al., 2007; Berdiel-Acer et al., 2021; Eaton and Timm, 2023). Consequently, TZB can lead to a marked reduction in left ventricular ejection fraction (LVEF) and an increased risk of HF (Choksey and Timm, 2021; Eaton and Timm, 2023; Bowles et al., 2012; Lin et al., 2021). The underlying mechanisms, particularly non-HER2 signaling-mediated effects and potential sex-based differences, are poorly characterized in TZB-induced chronic cardiotoxicity.

The sequence homology between human HER2 and rodent ErbB2 is approximately 84.4%, with 22 amino acid differences in the extracellular domain IV (Le et al., 2022). This region is crucial for TZB binding, and these differences may affect the antibody’s efficacy in rodent models (Le et al., 2022; Pegram and Ngo, 2006). Therefore, in rodent models, cardiotoxicity may be attributable to additional direct interactions of TZB with CMs, immune-mediated mechanisms, and off-target effects (Kitani et al., 2019; Rodrigues et al., 2024; Bartolo et al., 2025; Mohan et al., 2018). Possible explanations include (i) non-target toxicity from the drug or Fc receptor-mediated clearance in certain tissues (Liu et al., 2026; Mohan et al., 2018); (ii) TZB-induced immune responses like antibody-dependent cellular cytotoxicity (ADCC) (Bartolo et al., 2025); and (iii) off-target structural remodeling or disruption of compensatory cellular pathways, involving inflammation driven by oxidative stress, reduced antioxidant capacity, and altered cardiac metabolism (Eaton and Timm, 2023; Laird-Fick et al., 2020).

Disruption of cellular energy balance by TZB can lead to short- and long-term complications in cardiac cells. During glycolysis and the tricarboxylic acid cycle, nicotinamide adenine dinucleotide (NAD^+^) serves as the primary electron carrier. Furthermore, it is a precursor of *de novo* NADP^+^/NADPH synthesis, which plays a crucial role in anabolic and redox reactions (Chandel, 2021; Walker and Tian, 2024). Previous studies also linked the altered NAD^+^/NADH redox homeostasis to impaired cardiac function and consequential HF (Chandel, 2021). NAD^+^ is the end product of the main Trp catabolic pathway, i.e., the kynurenine pathway (Yang et al., 2024). The imbalance of Trp metabolites might contribute to the development of cardiovascular diseases. On one hand, several metabolites in the kynurenine pathway have antioxidant effects (e.g., xanthurenic acid) (Melhem and Taleb, 2021). On the other hand, several biologically active components of the kynurenine pathway (e.g., kynurenine and quinolinic acid) are associated with oxidative stress, inflammation, immune responses, mitochondrial dysfunction, and metabolic shifts that can contribute to the development and progression of HF (Grishanova and Perepechaeva, 2024; Hayes et al., 2023; Castro-Portuguez and Sutphin, 2020). However, their role and potential sex-dependent differences in TZB-induced chronic cardiotoxicity have not been described yet.

Therefore, we aimed to characterize alterations in LV concentrations of selected Trp metabolites in a rat model of TZB-induced chronic cardiotoxicity in both sexes.

## 2 Methods

### 2.1 Ethics approval

This investigation conformed to the EU Directive 2010/63/EU. It was approved by the regional Animal Research Ethics Committee of Csongrád County (project license number: X./440/2024, dated 26 March 2024) and the University of Szeged in Hungary.

### 2.2 Animals

A total of 12 male (300–400 g) and 14 female (200–260 g) Wistar-Hannover rats (8–10 weeks old, Charles-Rivers Laboratories, Germany) were housed in individually ventilated cages (Tecniplast Sealsafe IVC system, Buguggiate, Italy) under a 12/12-h light/dark cycle in a temperature-controlled room (22 °C ± 2 °C; relative humidity 55% ± 10%) with access to standard rat chow and tap water *ad libitum*.

### 2.3 Experimental setup

**FIGURE 1:**
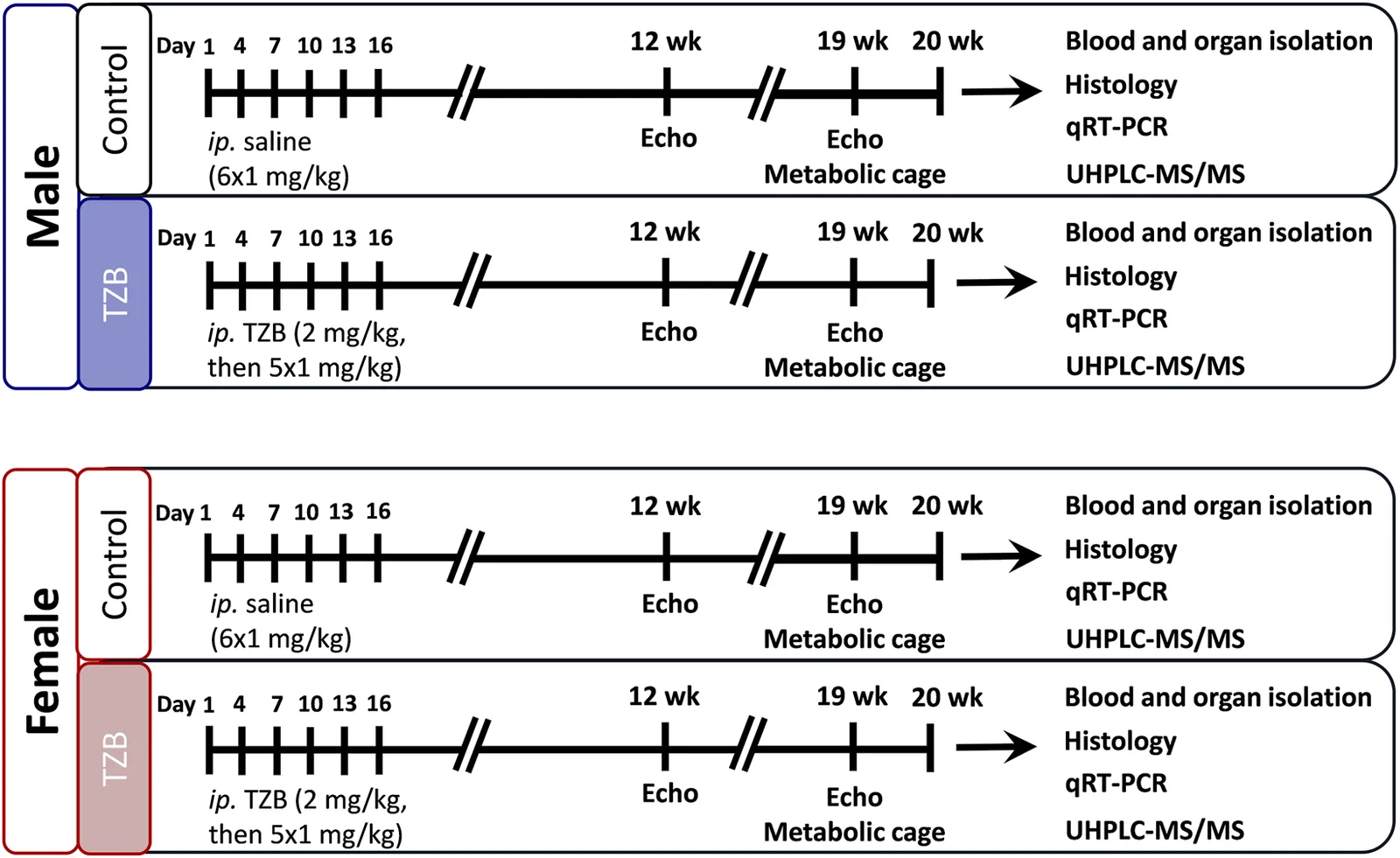
Protocol figure. Male and female Wistar-Hannover rats were divided into 2-2 groups: control treated with physiological saline (6 × 1 mL/kg, i.p.), and TZB (on the first day 2 mg/kg, then on days 4, 7, 10, 13, and 16, 1 mg/kg, i.p.) groups. At weeks 12 and 19, echocardiography was performed to assess cardiac function and morphology. Then, animals were placed in metabolic cages for 24 h to collect urine. At week 20, rats were anesthetized with sodium pentobarbital, and the abdominal cavity was opened to collect blood from the aorta. Then, under anesthesia, the hearts, lungs, and tibias were excised and washed in a calcium-free Krebs-Henseleit solution to remove the residual blood. The mid-papillary parts of the left ventricles were fixed in 4% buffered formalin for histological analysis, and the apical parts of the left ventricles were freshly frozen in liquid nitrogen and stored at −80 °C until further biochemical measurements, including RT-qPCR and ultra-high performance liquid chromatography-tandem mass spectrometry (UHPLC-MS/MS).

After 1 week of acclimatization, male and female animals were randomized into two control and two TZB-treated groups based on their body weight (n = 6–7, Figure 1). Control rats received saline (1 mL/kg, i.p.), and animals in the TZB-treated group received trastuzumab (at the first day, 2 mg/kg, i.p., then at days 4, 7, 10, 13, and 16, 1 mg/kg (i.e., 7 mg/kg cumulative dose), Ontruzant®, Teva Pharmaceutical Industries Ltd., Debrecen, Hungary, Figure 1). At weeks 12 and 19, echocardiography was performed to assess cardiac function and morphology. Then, animals were placed in metabolic cages (Tecniplast Metabolic Cage System, Buguggiate, Italy) for 24 h to collect urine. At week 20, rats were anesthetized with sodium pentobarbital (Euthasol; 40 mg/kg, ip.; Produlab Pharma b.v., Raamsdonksveer, Netherlands), and the abdominal cavity was opened to collect 1.0–1.5 mL of blood from the aorta. Then, rats were euthanized by overdosing on sodium pentobarbital (100 mg/kg, i.p.). The hearts, lungs, and tibias were excised and washed in a calcium-free Krebs-Henseleit solution to remove the residual blood. The total heart, LV, and lung weights were measured, and the left ventricles were transversely sectioned. The mid-papillary parts were fixed in 4% buffered formalin for histological analysis, and the apical parts of the left ventricles were freshly frozen in liquid nitrogen and stored at −80 °C until further biochemical measurements, including qRT-PCR and ultra-high performance liquid chromatography-tandem mass spectrometry (UHPLC-MS/MS, Figure 1).

### 2.4 Transthoracic echocardiography (TTE)

At weeks 12 and 19, cardiac morphology and function were assessed by TTE, as previously described (Sárközy et al., 2023), to monitor the development of TZB-induced chronic cardiotoxicity. Briefly, rats were anesthetized with 2% isoflurane (Forane, Aesica, Queenborough Limited, Queenborough, United Kingdom). Two-dimensional B-mode, M-mode, Doppler, tissue Doppler, and four-chamber-view images were performed by the criteria of the American Society of Echocardiography with a Vivid IQ ultrasound system (General Electric Medical Systems, New York, NY, United States) using a phased array 5.0–11 MHz transducer (General Electric 12S-RS probe, General Electric Medical Systems, New York, NY, United States). Data from three consecutive heart cycles were analyzed (EchoPac Dimension v201, General Electric Medical Systems, United States) by an experienced investigator in a blinded manner. The mean values of three measurements were calculated and used for statistical evaluation. Systolic and diastolic septal and posterior wall thicknesses were obtained from the parasternal long-axis view at the level of the mitral valve. The LV internal diameters at the end of systole and diastole (LVESD and LVEDD, respectively) were measured using M-mode echocardiography from the long-axis view between the endocardial borders. The left ventricular end-diastolic volume (LVEDV) and left ventricular end-systolic volume (LVESV) were calculated on four-chamber view images delineating the endocardial borders in diastole and systole. The stroke volume (SV) was calculated as the difference between LVEDV and LVESV. Ejection fraction (EF) was calculated using the formula (SV/LVEDV) × 100. Cardiac output (CO) was calculated as the product of SV and heart rate (HR). HR was calculated using pulsed-wave Doppler images. Diastolic function was assessed using pulsed-wave Doppler across the mitral valve and tissue Doppler imaging on the septal mitral annulus from the apical four-chamber view. Early (E) mitral inflow and septal mitral annulus velocity (e’) were used as an indicator of the diastolic function.

### 2.5 Urine laboratory parameters

At week 19, animals were placed into metabolic cages for 24 h to collect urine. Urine creatinine and protein levels were determined to assess the toxic effects of TZB on the kidneys (Figure 1). Urine creatinine and urine protein levels were measured by standard laboratory methods as described previously (Sárközy et al., 2021; Kovács et al., 2021).

### 2.6 Serum laboratory parameters

At week 20, serum albumin concentration was measured by a colorimetric assay (Hoffmann-La Roche Ltd., Switzerland) using bromocresol green (BCG) as an anionic dye that binds to albumin. The color intensity of the blue-green color is directly proportional to the albumin concentration in the sample and is measured photometrically. Serum urea and creatinine levels were quantified using a kinetic UV spectrophotometric method with urease and glutamate dehydrogenase enzymes, following Jaffe’s method as previously described (Sárközy et al., 2023; Kovács et al., 2021). The reagents and the platform analyzers were from Roche Diagnostics (Hoffmann-La Roche Ltd., Switzerland). Creatinine clearance, an indicator of renal function, was calculated according to the standard formula (urine creatinine concentration [μM] × urine volume for 24 h [mL])/(serum creatinine concentration [μmol/L] × 24 × 60 min). Urine volume and urine creatinine concentrations were measured at week 19 (Sárközy et al., 2023; Kovács et al., 2021).

### 2.7 Histology

Formalin-fixed paraffin-embedded subvalvular areas of the LV were cut transversally into 5-μm sections and stained with hematoxylin-eosin (HE) or picrosirius red/fast green (PSFG) stainings, as described previously [26,28]. Histological slides were scanned with a Pannoramic Midi II scanner (3D-Histech, Budapest, Hungary). Digital slide processing was performed using SlideViewer version 2.6 (3D-Histeech, Budapest, Hungary). Representative HE- and PSFG-stained slides were captured in Panoramic Viewer 1.15.4 (3D-Histech, Budapest, Hungary; https://old.3dhistech.com/pannoramic_viewer). On the digital HE images, CM cross-sectional areas (100/CM) were evaluated using the Biology Image Analysis Software (BIAS 1.0, Single-Cell Technologies Ltd., Szeged, Hungary, https://single-cell-technologies.com/bias/), as described previously (Sárközy et al., 2023; Kovács et al., 2021). LV interstitial fibrosis was assessed on PSFG slides using an in-house-developed program as described previously (Sárközy et al., 2023; Kovács et al., 2021).

### 2.8 Transcription profiling by qRT-PCR in left ventricular tissue samples

Quantitative RT-PCR was performed with gene-specific primers to monitor LV mRNA expression, as described previously (Kovács et al., 2021). RNA was isolated using Qiagen RNeasy Fibrous Tissue Mini Kit (Qiagen, Hilden, Germany) and quantified by NanoDrop One Microvolume UV-VIS spectrophotometer (Thermo Fisher Scientific Inc., Waltham, MA, United States). Then, 100 μg of total RNA was reverse-transcribed using the iScript cDNA Synthesis Kit (Bio-Rad Laboratories Inc., Hercules, CA, United States). The cDNAs were analyzed in technical duplicates using 10 μL reaction volumes. Specific primers (*Acox1*: peroxisomal acyl-coenzyme A oxidase 1, #qRnoCIP0027493; *Cpt1a*: carnitine O-palmitoyl transferase 1, liver isoform, #qRnoCIP0031435; *Ctgf*: connective tissue growth factor, #qRnoCED0001593; *Gapdh*: glyceraldehyde-3-phosphate dehydrogenase, #qRnoCID0057018; *Il1b*: interleukin-1 beta precursor, #qRnoCID0004680; *Il6*: interleukin-6, #qRnoCID0053166; *Ldha*: lactate dehydrogenase A chain, #qRnoCED0006780; *Myh6*: α-myosin heavy chain, #qRnoCID0001766; *Myh7*: β-myosin heavy chain, #qRnoCED0001215; *Nos2*: inducible nitric oxide synthase, #qRnoCID0004849; *Nos3*: endothelial nitric oxide synthase, #qRnoCID0005021; *Pkm*: pyruvate kinase isozymes M1/M2, #qRnoCID0002639; *Slc2a1*: solute carrier family 2 member 1 (i.e., glucose transporter 1), #qRnoCEP0025043; and *Slc2a4*: solute carrier family 2 member 4 (i.e., glucose transporter 4), #qRnoCIP0023827) and SsoAdvanced™ Universal SYBR® Green Supermix (all from BioRad Laboratories Inc., Hercules, CA, United States) were applied according to the manufacturer’s instructions using a BioRad CFX-96 machine with the accompanying BioRad CFX Manager (BioRad Laboratories Inc., Hercules, CA, United States) software for cycle threshold value analysis. Gene expression was calculated using the relative standard curve method. Peptidyl-prolyl cis-trans isomerase A (*Ppia*, #qRnoCID0056995, BioRad Laboratories Inc., Hercules, CA, United States) was used as a housekeeping control gene for normalization.

### 2.9 Left ventricular metabolite concentration measurements by UHPLC-MS/MS

The LV samples were prepared, and a targeted panel of metabolites spanning tryptophan metabolism (kynurenine, serotonin, and indole pathways), redox homeostasis, nitric oxide–related cofactors, one-carbon metabolism, energy metabolism, and cyclic nucleotide signaling was quantified by ultra-high-performance liquid chromatography–tandem mass spectrometry (UHPLC-MS/MS) according to previously published methodologies (Galla et al., 2021a; Galla et al., 2021b; Polyák et al., 2023). MRM transition of indoxyl sulfate (INS) was 211.9/131.9 using −50 V as the declustering potential and −25 V as the collision energy; retention time: 11.48 min. MRM transition of asymmetric dimethylarginine (ADMA) was 203.2/46.0 using 50 V as the declustering potential and 30 V as the collision energy; retention time: 1.00 min. MRM transition of symmetric dimethylarginine (SDMA) was 203.2/172.0 using 50 V as the declustering potential and 20 V as the collision energy; retention time: 1.06 min. MRM transition of reduced glutathione (GSH) was 308.2/179.1 using 50 V as the declustering potential and 14 V as the collision energy; retention time: 1.76 min. MRM transition of oxidized glutathione (GSSG) was 614.4/485.1 using 50 V as the declustering potential and 17 V as the collision energy; retention time: 2.58 min. MRM transition of NAD^+^ was 664.2/136.1 using 85 V as the declustering potential and 50 V as the collision energy; retention time: 2.00 min. MRM transition of lactate was 88.9/43.0 using −50 V as the declustering potential and −15 V as the collision energy; retention time: 1.70 min. MRM transition of pyruvate was 86.9/59.0 using −50 V as the declustering potential and −11 V as the collision energy; retention time: 1.30 min. MRM transition of indole-3-lactic acid was 206.1/188.1 using 50 V as the declustering potential and 12 V as the collision energy; retention time: 13.00 min. MRM transition of indole-3-propionic acid was 190.1/130.1 using 50 V as the declustering potential and 19 V as the collision energy; retention time: 13.00 min. MRM transition of indole-3-carboxaldehyde was 146.1/118.1 using 50 V as the declustering potential and 19 V as the collision energy; retention time: 12.40 min. MRM transition of cyclic guanosine monophosphate (cGMP) was 346.1/135.0 using 50 V as the declustering potential and 52 V as the collision energy; retention time: 5.00 min. MRM transition of biopterin (BIO) was 238.1/178.1 using 30 V as the declustering potential and 26 V as the collision energy; retention time: 2.54 min. MRM transition of dihydrobiopterin (BH2) was 240.2/196.1 using 30 V as the declustering potential and 16 V as the collision energy; retention time: 2.30 min. MRM transition of 5′-methyltetrahydrofolic acid (Me5THF) was 460.3/313.2 using 50 V as the declustering potential and 21 V as the collision energy; retention time: 7.27 min.

### 2.10 Statistical analysis

All analyses were performed with GraphPad Prism Software (version 10.3.1, GraphPad Software Inc., Boston, MA, United States). The normality of data distribution was tested by the Shapiro-Wilk test. Two-way analysis of variance (ANOVA) was used to compare groups. When significant differences between groups were observed, the Holm-Sidak method was used for *post hoc* comparisons. All values are presented as mean ± S.E.M., and a p-value of <0.05 was accepted as statistically significant.

## 3 Results

### 3.1 TZB treatment resulted in reduced creatinine clearance only in females

There were no significant differences in serum albumin, urea, and creatinine concentrations, urine volume, urine protein, or creatine levels between the TZB-treated groups and the sex-matched control groups (Table 1). In contrast, creatine clearance was significantly lower in the female TZB-treated group than in the female control group, indicating renal dysfunction (Table 1). Notably, creatine clearance was markedly lower in females than in males, attributable to their smaller body size (Table 1).

**TABLE 1:** Laboratory parameters in response to trastuzumab (TZB) treatment in both sexes.

Parameter (unit)	CT ♂	TZB ♂	CT ♀	TZB ♀
Serum albumin (g/L)	36 ± 0.71	35 ± 0.59	43 ± 0.92^#^	41 ± 0.97^#^
Urine protein (mg/dL)	39 ± 9.79	39 ± 8.66	83 ± 17.0	88 ± 29.5
Serum urea (mmol/L)	6.88 ± 0.38	6.94 ± 0.15	7.16 ± 0.37	7.66 ± 0.26^#^
Serum creatinine (μmol/L)	40 ± 2.28	36 ± 2.94	36 ± 2.55	41 ± 1.95
Urine creatinine (µmol/L)	4,865 ± 823	4,455 ± 1,094	4,254 ± 394	3,436 ± 420
Urine volume (mL)	31 ± 4.23	36 ± 6.05	28 ± 5.81	26 ± 3.57
Creatinine clearance	2.12 ± 0.12	2.53 ± 0.28	1.46 ± 0.09^#^	1.19 ± 0.07*^#^

Values are presented as mean ± S.E.M., *p < 0.05 control vs. TZB group, #p < 0.05 males vs. females, n = 6-7, One-Way ANOVA, Holm-Sidak *post hoc* test. CT: control; TZB: trastuzumab.

### 3.2 TZB treatment reduced the LV wall thicknesses in both sexes at the endpoint

**FIGURE 2:**
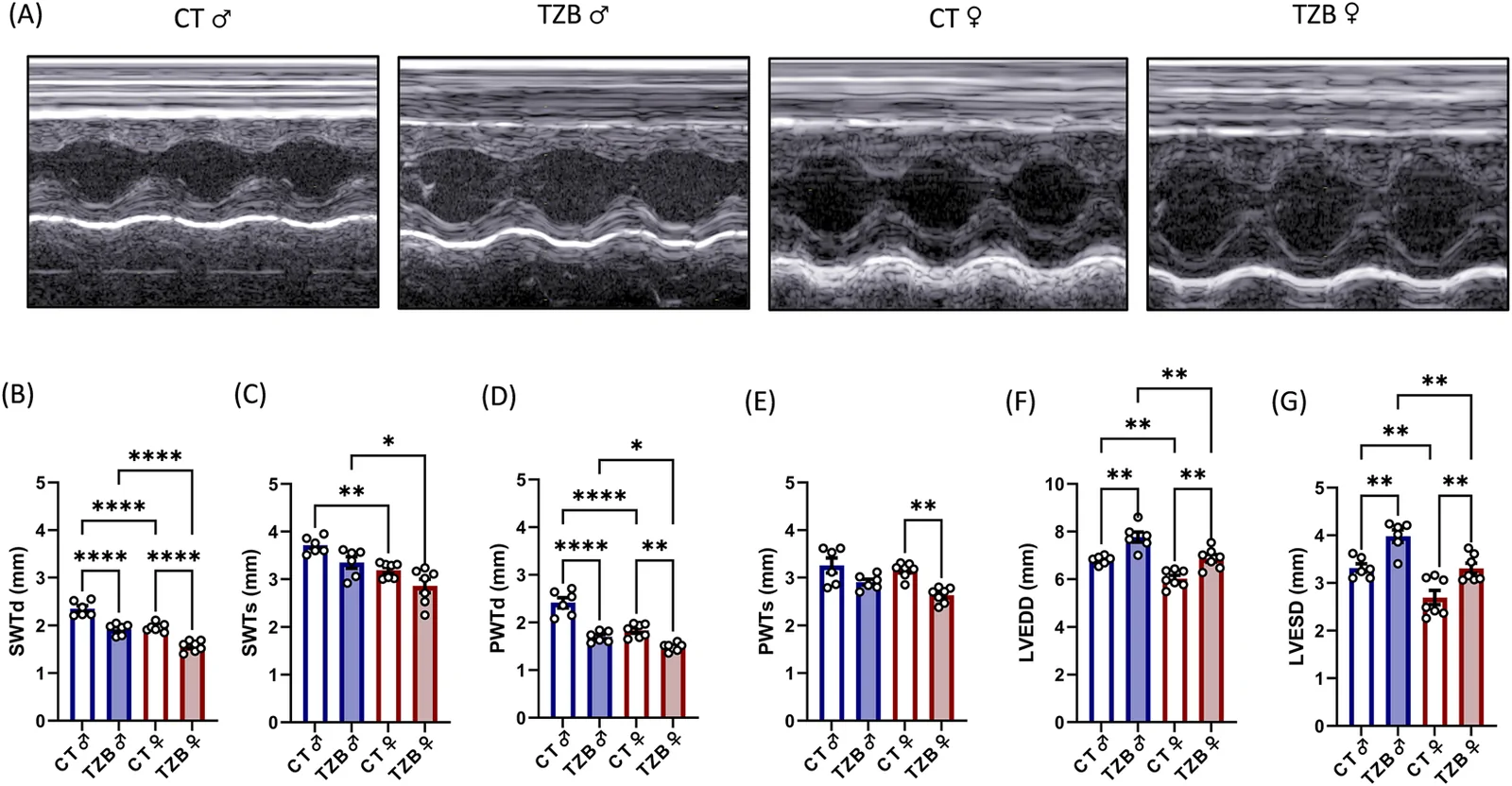
Left ventricular morphologic alterations in response to trastuzumab (TZB) treatment assessed by echocardiography in both sexes. **(A)** Representative M-mode images, **(B)** septal wall thickness in diastole (SWTd), **(C)** septal wall thickness in systole (SWTs), **(D)** posterior wall thickness in diastole (PWTd), **(E)** posterior wall thickness in systole (PWTs), **(F)** left ventricular end-diastolic diameter (LVEDD), and **(G)** left ventricular end-systolic diameter (LVESD). Values are presented as mean ± S.E.M., *p < 0.05, **p < 0.01, ***p < 0.001, ****p < 0.0001, n = 6-7, Two-Way ANOVA, Holm-Sidak *post hoc* test. CT: Control and TZB: trastuzumab. Each circle corresponds to one individual data point.

**FIGURE 3:**
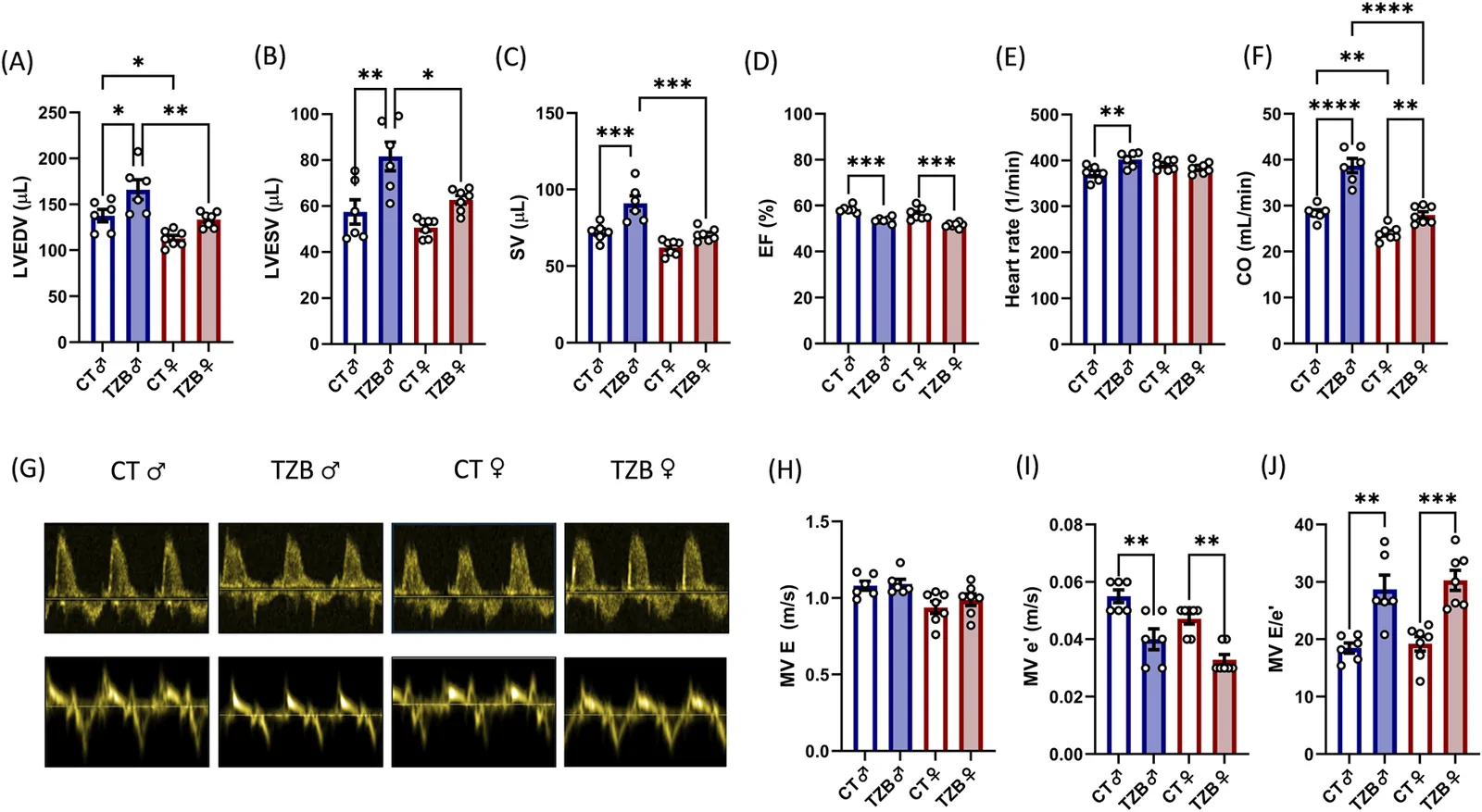
Left ventricular functional alterations in response to trastuzumab (TZB) treatment assessed by echocardiography in both sexes. **(A)** Left ventricular end-diastolic volume (LVEDV), **(B)** left ventricular end-systolic volume (LVESV), **(C)** stroke volume (SV), **(D)** ejection fraction (EF), **(E)** heart rate (HR), **(F)** cardiac output (CO), **(G)** representative Doppler and tissue Doppler images, **(H)** peak early diastolic mitral inflow velocity **(E)**, **(I)** early diastolic mitral annular velocity (e’), and **(J)** E/e’. Values are presented as mean ± S.E.M., *p < 0.05, **p < 0.01, ***p < 0.001, ****p < 0.0001, n = 6-7, Two-Way ANOVA, Holm-Sidak *post hoc* test. CT: Control and TZB: trastuzumab. Each circle corresponds to one individual data point.

At weeks 12 and 19, transthoracic echocardiography (TTE) was performed to assess LV morphological and functional changes in response to TZB (Supplementary Figures 1A–1G, 2A–2J; Figures 2A–G, and Figures 3A–3J). At week 12, there were no significant differences in LV wall thicknesses and diameters between the TZB-treated groups and their sex-matched control groups (Supplementary Figures 1A–1G). However, LV wall thicknesses and diameters were significantly lower in females than in males, independent of TZB treatment, due to the smaller body size of females (Supplementary Figures 1A–1G).

At week 19, the systolic and the diastolic septal and the diastolic posterior wall thicknesses were significantly thinner in both TZB-treated groups compared to the sex-matched control groups (Figures 2A–E). The male TZB-treated group showed a decreasing tendency (p = 0.056) in the systolic posterior wall thickness compared to the sex-matched control group (Figure 2E). The decreased septal and posterior wall thickness resulted in markedly increased LVEDD and LVESD in both TZB-treated groups compared to their sex-matched control groups (Figures 2F,G). Independent of TZB treatment, the septal and posterior walls in diastole, the septal wall in systole, and the LV diameters were significantly reduced compared with the values of the corresponding male groups due to the smaller body size of females (Figures 2A–D,F,G). Indeed, irrespective of TZB treatment, body weight, organ weight, and tibia length were markedly lower in the female groups than in the male groups (Table 2).

**TABLE 2:** Body weights and organ weights in response to trastuzumab (TZB) treatment in both sexes.

Parameter (unit)	CT ♂	TZB ♂	CT ♀	TZB ♀
Body weight at the beginning (g)	343 ± 16.45	341 ± 13.13	233 ± 5.28^#^	240 ± 6.99^#^
Body weight at the endpoint (g)	485 ± 20.89	521 ± 28.92	287 ± 8.75^#^	291 ± 3.04^#^
Heart weight (mg)	1,102 ± 56.87	1,113 ± 49.23	743 ± 12.95^#^	736 ± 7.89^#^
Left ventricular weight (mg)	900 ± 41.95	887 ± 49.23	604 ± 10.37^#^	601 ± 6.76^#^
Right ventricular weight (mg)	187 ± 23.28	205 ± 11.08	132 ± 9.63^#^	130 ± 1.68^#^
Lung weight (mg)	1,522 ± 39.47	1,526 ± 69.29	1,180 ± 30.06^#^	1,163 ± 12.10^#^
Kidney weight (mg)	1,313 ± 73.70	1,251 ± 61.48	848 ± 27.43^#^	858 ± 13.24^#^
Tibia length (mm)	42 ± 0.30	43 ± 0.58	38 ± 0.50^#^	37 ± 0.20^#^

Values are presented as mean ± S.E.M., *p < 0.05 control vs. trastuzumab group, #p < 0.05 males vs. females, n = 6-7, Two-Way ANOVA, Holm-Sidak *post hoc* test. CT: control; TZB: trastuzumab.

### 3.3 TZB treatment resulted in diastolic and systolic dysfunction in both sexes

At week 13, there were no significant changes in LVEDV, LVESV, SV, CO, EF, and HR between the TZB-treated groups and their sex-matched control groups (Supplementary Figures 2A–2F). In accordance with the LV diameters, LVEDV, LVESV, SV, and CO were markedly lower in the females compared to the males, irrespective of TZB treatment (Supplementary Figures 2A–2C,2F). However, e’ was decreased in both TZB-treated groups compared to their sex-matched control groups, indicating diastolic dysfunction (Supplementary Figures 2G, 2I). In contrast, there were no marked changes in E velocity and the E/e’ between the groups (Supplementary Figures 2H, 2J).

At week 19, in consonance with the LV diameters, LVEDV, LVESV, and SV were markedly increased in the TZB-treated groups compared to the sex-matched control groups (Figures 3A–C). The EF was significantly reduced in both TZB-treated groups compared to the sex-matched control groups, indicating systolic dysfunction (Figure 3D). The heart rate was not significantly different among the groups (Figure 3E). CO was markedly increased in the TZB-treated male group compared with the sex-matched control group (Figure 3F). In the TZB-treated groups, the LVEDV, LVESV, SV, and CO were markedly lower in females than in males (Figures 3A–C,F).

There were no significant differences in the E velocity among the groups (Figures 3G,H). The e’ velocity was markedly decreased, and the E/e’ was significantly higher in both TZB-treated groups compared to the sex-matched control groups, indicating diastolic dysfunction (Figures 3G,I,J).

### 3.4 Cardiomyocyte hypertrophy and interstitial fibrosis developed in both sexes in response to TZB treatment

**FIGURE 4:**
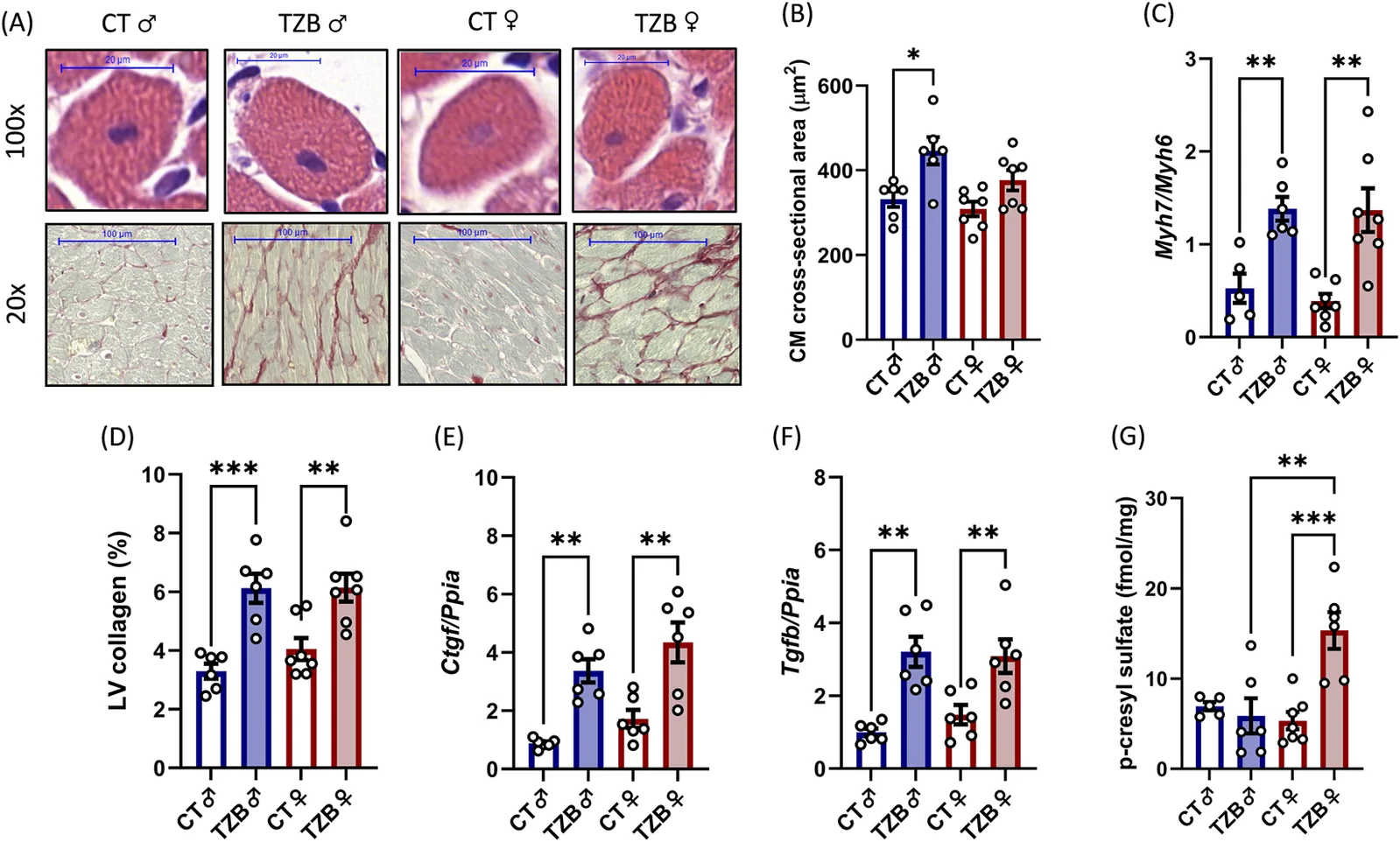
The effects of TZB and sex on cardiomyocyte hypertrophy, interstitial fibrosis, and related gene expressions and metabolite levels. **(A)** Representative histologic sections stained with hematoxylin-eosin (HE, 100x) and picrosirius red and fast green (PSFG, 20x), **(B)** cardiomyocyte (CM) cross-sectional area, **(C)** beta-myosin heavy chain to alpha-myosin heavy chain ratio (*Myh7/Myh6*), **(D)** left ventricular (LV) collagen content, LV expression of **(E)** connective tissue growth factor (*Ctgf*), **(F)** transforming growth factor beta (*Tgfb*), and **(G)** LV concentration of p-cresyl sulfate. Values are presented as mean ± S.E.M., *p < 0.05, **p < 0.01, ***p < 0.001, n = 6-7, Two-Way ANOVA, Holm-Sidak *post hoc* test. CT: Control and TZB: trastuzumab. Each circle corresponds to one individual data point.

On the HE-stained slides, CM cross-sectional areas were significantly increased in the male TZB-treated group compared to the sex-matched control group, indicating compensatory CM hypertrophy (Figures 4A,B); however, there were no marked changes between the female TZB-treated and control groups (Figures 4A,B). In addition, a molecular marker of CM hypertrophy, the ratio of cardiac myosin heavy chain beta (*Myh7*) and alpha (*Myh6*) isoforms, was markedly increased in both TZB-treated groups (Figure 4C).

On the PSFG-stained slides, the LV collagen content was significantly increased in both TZB-treated groups compared to the sex-matched control groups, indicating interstitial fibrosis (Figure 4D). Moreover, several fibrosis-associated markers, including *Ctgf* and *Tgfb* were significantly overexpressed in the left ventricles of the TZB-treated groups compared to the sex-matched control groups (Figures 4E,F). Interestingly, the concentration of the hypertrophy- and fibrosis-inducing uremic toxin, i.e., para-cresyl-sulfate, was markedly increased in the LV of TZB-treated females compared to that in control females in consonance with the reduced creatinine clearance in the TZB-treated females (Figure 4G; Table 1).

### 3.5 TZB treatment resulted in elevated LV expression of inflammatory markers in both sexes

**FIGURE 5:**
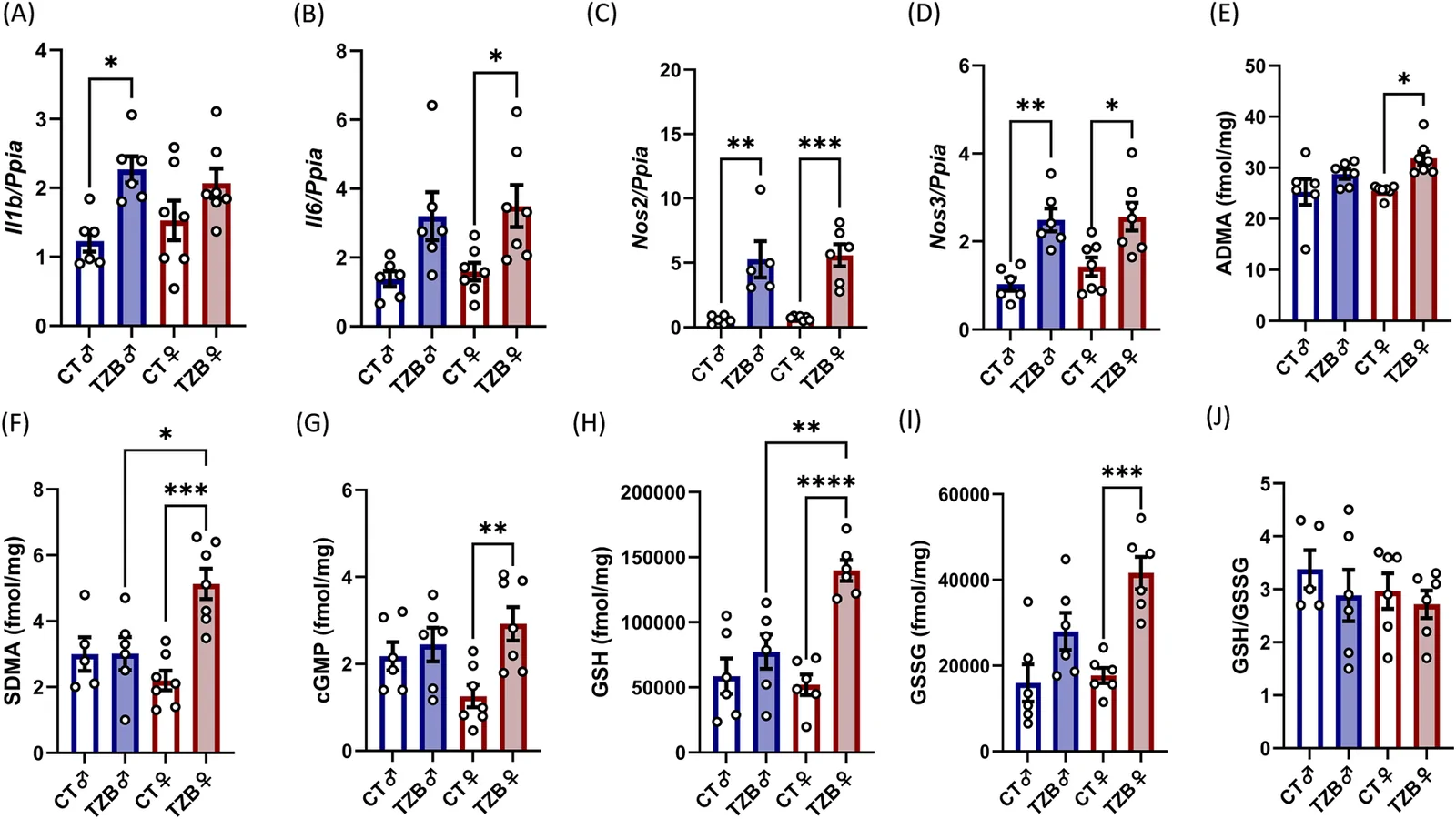
Changes in the left ventricular gene expressions and metabolite levels related to nitro-oxidative stress and inflammation in response to TZB treatment in both sexes. **(A)** Interleukin-1 (*Il1b*), **(B)** interleukin-6 (*Il6*), **(C)**, inducible nitric oxide synthase (*Nos2*), **(D)** endothelial nitric oxide synthase (*Nos3*), **(E)** asymmetric dimethylarginine (ADMA), **(F)** symmetric dimethylarginine (SDMA), **(G)** cyclic guanosine monophosphate (cGMP), **(H)** reduced glutathione (GSH), **(I)** oxidized glutathione (GSSG) and **(J)** reduced glutathione to oxidized glutathione ratio (GSH/GSSG). Values are presented as mean ± S.E.M., *p < 0.05, **p < 0.01, ***p < 0.001, n = 5-7, One-Way ANOVA, Holm-Sidak *post hoc* test. CT: Control and TZB: trastuzumab. Each circle corresponds to one individual data point.

The inflammatory marker *Il1b* was significantly overexpressed in the LV samples of the male TZB-treated group, whereas *Il6* was markedly overexpressed in the female TZB-treated group compared to the sex-matched control groups, respectively (Figures 5A,B). Indeed, inducible nitric oxide synthase (*Nos2*), which is associated with inflammation, was markedly overexpressed in both TZB-treated groups compared to the sex-matched control groups (Figure 5C).

### 3.6 TZB treatment increased the LV levels of several eNOS/cGMP pathway components in females only

The endothelial nitric oxide synthase (*Nos3/*eNOS) was overexpressed in the left ventricles of both TZB-treated groups compared to the sex-matched control groups (Figure 5D). Concentrations of the endogenous eNOS inhibitors and uremic toxins, i.e., asymmetric and symmetric dimethylarginines (ADMA and SDMA, respectively), were markedly higher in the LV TZB-treated female group as compared with the female control group (Figures 5E,F). Moreover, the levels of the eNOS cofactors tetrahydrobiopterin (BH4), its oxidized form biopterin (BIO), its partially oxidized form, dihydrobiopterin (BH2) and 5′-methyltetrahydrofolic acid (Me5THF), as well as the second messenger cGMP, were significantly increased in the left ventricles of the female TZB-treated group compared to the female control group, probably as a compensation to higher ADMA and SDMA levels (Supplementary Figures 3A–3C; Figures 5E–G).

### 3.7 The GSH/GSSG ratio did not change in response to TZB treatment in both sexes

There were no significant differences in the LV levels of reduced and oxidized glutathione (GSH and GSSG, respectively) and their ratio between the male TZB-treated and control groups (Figures 5H–J). In contrast, GSH and GSSG were markedly elevated in the TZB-treated females as compared to the control females (Figures 5H,I). However, the GSH/GSSG ratio did not increase in the female TZB-treated group compared with the female control group (Figure 5J).

### 3.8 TZB treatment induced LV overexpression of *Nrg1* in females and *Erbb4* and *Mtor* overexpression in both sexes

*Nrg1* was overexpressed in the left ventricles of the female TZB-treated group compared to the sex-matched control group (Supplementary Figure 4A). There was no significant difference in the LV *Erbb2* expression among the groups (Supplementary Figure 4B). In response to the TZB treatment, *Erbb4* was overexpressed in both sexes (Supplementary Figure 4C). Furthermore, TZB treatment induced marked overexpression of the cell growth- and metabolism-regulating mammalian target of rapamycin (*Mtor*) gene in the LV in both sexes (Supplementary Figure 4D).

### 3.9 TZB treatment reduced the expression of several genes involved in glycolysis in the LV in both sexes

**FIGURE 6:**
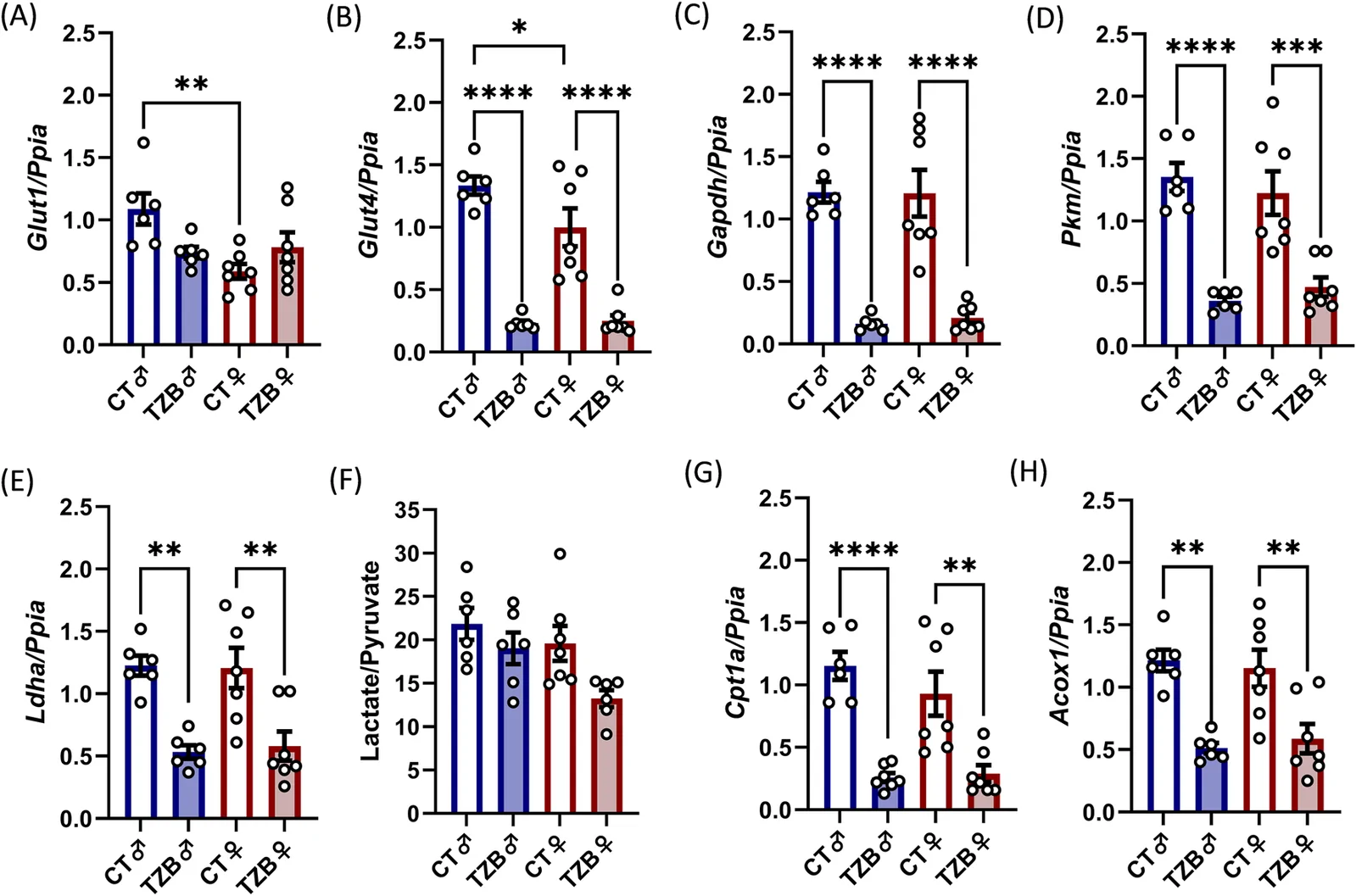
Changes in the left ventricular gene expression and metabolite concentrations related to glucose and fatty acid metabolism in response to TZB treatment in both sexes. **(A)** Glucose transporter-1 (*Slc2a1*), **(B)** glucose transporter 4 (*Slc2a4*), **(C)** glyceraldehyde-3-phosphate dehydrogenase (*Gapdh*), **(D)** pyruvate kinase (*Pkm*), **(E)** lactate dehydrogenase-A (*Ldha*), **(F)** lactate/pyruvate ratio, **(G)** carnitine O-palmitoyltransferase-1α (*Cpt1α*), and **(H)** peroxisomal acyl-coenzyme A oxidase-1 (*Acox1*). Values are presented as mean ± S.E.M., *p < 0.05, **p < 0.01, ***p < 0.001, ****p < 0.0001, n = 5-7, Two-Way ANOVA, Holm-Sidak *post hoc* test. CT: Control and TZB: trastuzumab. Each circle corresponds to one individual data point.

There was no significant difference in the LV expression of the insulin-independent glucose transporter 1 (*Slc2a1*) among the groups (Figure 6A). The insulin-dependent glucose transporter 4 (*Slc2a4*) was significantly repressed in both TZB-treated groups compared to the sex-matched control groups (Figure 6B). The LV expressions of glyceraldehyde-3-phosphate dehydrogenase (*Gapdh*), pyruvate-kinase (*Pkm*), and lactate-dehydrogenase (*Ldha*) were markedly decreased in both TZB-treated groups compared to the sex-matched control groups (Figures 6C–E). The lactate/pyruvate ratio showed a decreasing tendency (p = 0.076) only in the TZB-treated females as compared to the control females (Figure 6F).

### 3.10 TZB treatment reduced the LV expression of several genes involved in β-oxidation in both sexes

The LV expressions of carnitine palmitoyltransferase-1α (*Cpt1α*) and peroxisomal acyl-coenzyme A oxidase 1 (*Acox1*) were significantly decreased in both TZB-treated groups as compared to those in the sex-matched control groups (Figures 6G,H).

### 3.11 TZB treatment induced sex-divergent changes in the LV levels of several Trp metabolites

**FIGURE 7:**
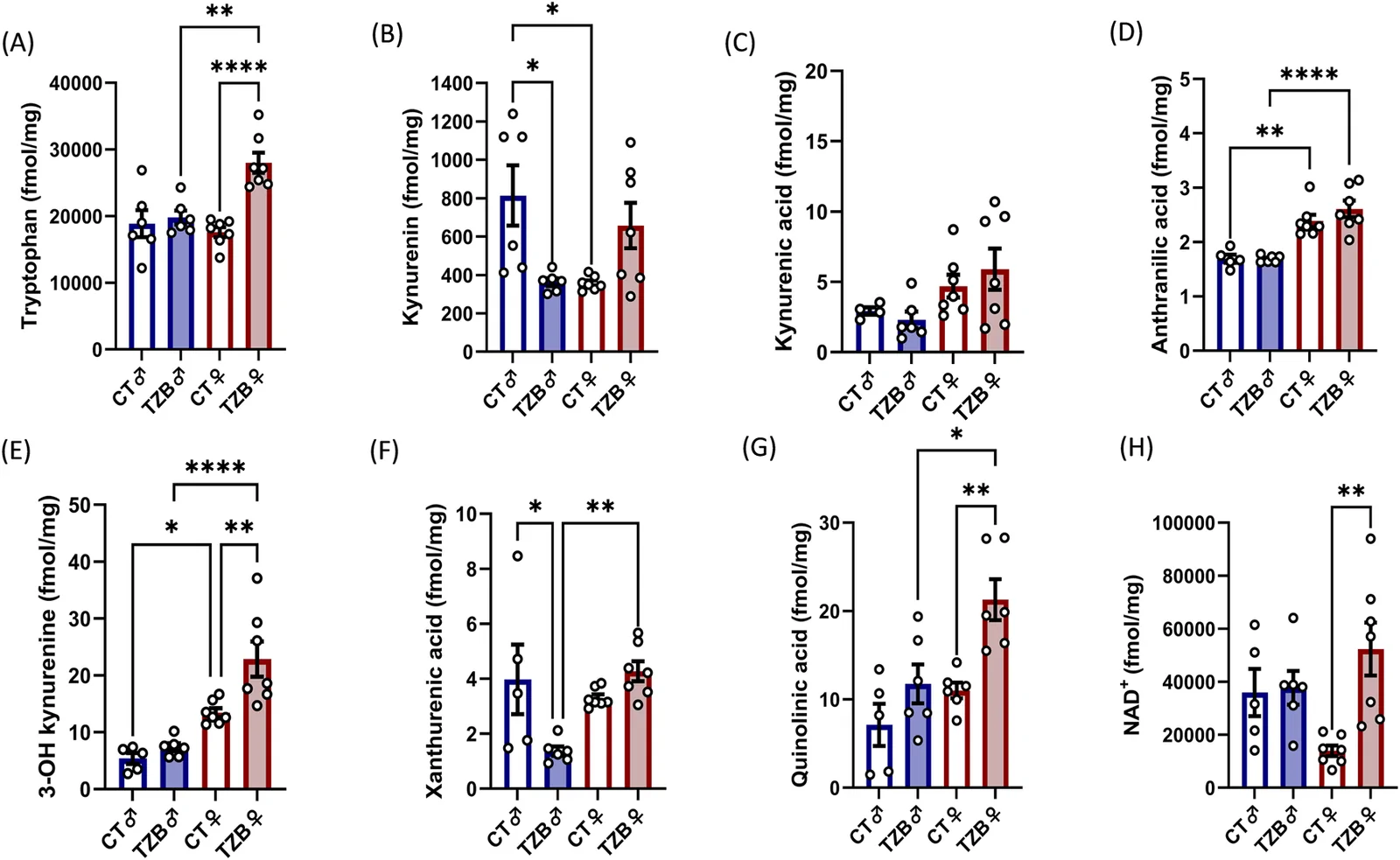
Changes in the left ventricular levels of tryptophan and its metabolites in the kynurenine pathway in response to TZB treatment in both sexes. **(A)** Tryptophan (Trp), **(B)** kynurenine, **(C)** kynurenic acid, **(D)** anthranilic acid, **(E)** 3-hydroxykynurenine (3-OH kynurenine), **(F)** xanthurenic acid, **(G)** quinolinic acid, and **(H)** nicotinamide-adenine-dinucleotide (NAD^+^). Values are presented as mean ± S.E.M., *p < 0.05, **p < 0.01, ***p < 0.001, ****p < 0.0001, n = 5-7, Two-Way ANOVA, Holm-Sidak *post hoc* test. CT: Control and TZB: trastuzumab. Each circle corresponds to one individual data point.

Trp levels in the left ventricles were significantly higher in the TZB-treated female group compared to the sex-matched control and the TZB-treated male group (Figure 7A). The concentration of kynurenine was markedly lower in the left ventricles in the TZB-treated male group as compared to the sex-matched control group (Figure 7B). There were no significant differences in LV kynurenic acid concentration among the groups (Figure 7C). The LV anthranilic acid and 3-hydroxykynurenine concentrations were markedly higher in females compared to males, independently of TZB treatment (Figures 7D,E). Interestingly, 3-hydroxykynurenine concentrations were significantly higher in the female TZB-treated group than in the sex-matched control group (Figure 7E). The LV xanthurenic acid levels were markedly lower in the TZB-treated male group compared to the sex-matched control and TZB-treated female groups (Figure 7F). In response to the TZB treatment, LV quinolinic acid and NAD^+^ levels were markedly higher in the female TZB-treated group compared to the female control group (Figures 7G,H).

No significant differences were observed in the LV concentrations of serotonin (Supplementary Figure 5A). Indol-3-acetic acid concentration was markedly higher in the TZB-treated females as compared to that in the TZB-treated males (Supplementary Figure 5B). There were no significant differences in the LV levels of other Trp metabolites, including indol-3-lactic acid, 5-hydroxyindolacetic acid, indol-3-propionic acid, and indol-3-carboxaldehyde among the groups (Supplementary Figures 5C–5F).

## 4 Discussion

To our best knowledge, our exploratory and descriptive study is the first to investigate potential sex-based differences in TZB-induced chronic cardiotoxicity in a rat model. Here, we characterized TZB-induced chronic changes in markers of nitro-oxidative stress, inflammation, glucose, and fatty acid utilization, as well as several elements of the kynurenine and serotonin pathways in the left ventricles in both sexes.

Although clinical studies showed that the onset and severity of TZB-induced cardiotoxicity varied greatly and that the condition might be reversible upon discontinuation of therapy, some patients suffer from long-term, irreversible heart damage, leading to decreased LVEF and consequent HF (Eaton and Timm, 2023; Bowles et al., 2012; Lin et al., 2021). TZB is used in oncology patients mainly with breast and gastric cancer in real life. Notably, breast and gastric cancers are associated with an increased risk of cardiovascular morbidity and mortality, even in the absence of specific oncological therapy (Zhao et al., 2025; Guan et al., 2020). Unfortunately, our present study fails to capture how TZB interacts with the complex, immunosuppressive tumor microenvironment. Conducting preclinical studies in normal (healthy) rats can only provide data on general pharmacokinetics and tumor-independent toxicity. Several studies have investigated the direct cardiac effects of TZB in cancer patients so far (Mantarro et al., 2016; Telli et al., 2007). However, the pathomechanism of TZB-induced chronic cardiotoxicity remains incompletely understood, and limited data are available on potential sex-based differences in its development.

In rats, the effects of TZB are often studied at doses of 4 mg/kg or higher for 7–30 days to achieve measurable cardiac effects within a short follow-up period, resulting in a short-term cardiotoxicity model (Arinno et al., 2023; Khan et al., 2023; Mousa et al., 2022). In our present study, we administered a relatively low cumulative TZB dose of 7 mg/kg to investigate its long-term cardiotoxic effects, without early animal mortality. This prolonged follow-up period (i.e., 20 weeks) is a unique approach in animal models. Actually, many clinical studies have reported that the risk of chronic HF and the asymptomatic drop of LVEF is 1.6–5.7 fold higher in TZB-treated patients (especially after anthracycline administration and with preexisting cardiovascular risk factors) over a long-term follow-up (Lin et al., 2021). Fluctuating sex hormones, particularly estrogen, can significantly alter cardiovascular parameters, and high estrogen levels can influence the efficacy and degradation rate of TZB, thereby contributing to TZB resistance (Pegram et al., 2023; Montagna and Colleoni, 2019; Ocaña et al., 2006). However, patients receiving TZB therapy are primarily young women of reproductive age (between 30 and 45 years old) with breast cancer (Natsuhara and Chien, 2024). Accordingly, in our present study, our primary goal was to investigate whether TZB can induce chronic cardiotoxicity with a well-defined endpoint, such as fibrosis, in a rat model, independent of the estrous cycle.

At week 12, diastolic dysfunction started to develop in both TZB-treated groups as indicated by reduced e’. At the endpoint, TZB treatment resulted in LV wall thinning and chamber dilation, leading to reduced EF and diastolic dysfunction (i.e., decreased e’ and increased E/e’) in both sexes, consistent with human studies (Eaton and Timm, 2023; Bowles et al., 2012; Lin et al., 2021; Moghadam et al., 2025). Here, we demonstrated histological and molecular signs of LV fibrosis (i.e., *Tgf* and *Ctgf* overexpression) and CM hypertrophy (i.e., increased *Myh7/Myh6* ratio), which are well-known contributors to the development of systolic and diastolic dysfunction (Eaton and Timm, 2023; Bowles et al., 2012; Lin et al., 2021; Rochette et al., 2015; Zhu et al., 2025). We hypothesize that CM hypertrophy develops to compensate for cell loss and maintain cardiac pump function.

Increased nitro-oxidative stress and inflammation have already been confirmed as contributors to LVH and fibrosis in HF (Eaton and Timm, 2023; Rochette et al., 2015; Zou et al., 2002; Senderovic and Galijasevic, 2024). Indeed, the chronic upregulation of IL6- and IL1-mediated signaling has been reported to play a key role in the development of LVH and fibrosis in preclinical and clinical studies (Eaton and Timm, 2023; Lin et al., 2021). In line with this, a nitro-oxidative stress-associated marker (i.e., *Nos2* overexpression in both sexes) and molecular signs of inflammation (i.e., overexpression of *Il1* in males and *Il6* in females) were observed in our chronic TZB-induced cardiotoxicity model, as reported in previous preclinical studies (Eaton and Timm, 2023; Khan et al., 2023; Mousa et al., 2022). Notably, *Nos2* overexpression has been shown to induce further inflammation and nitro-oxidative stress (Rochette et al., 2015; Senderovic and Galijasevic, 2024). In our present study, *Nos3* was overexpressed in both TZB-treated groups, and cGMP levels were higher in TZB-treated females. It has been demonstrated that the depletion of the eNOS cofactor BH4 or the elevation of its oxidized form, BH2, may lead to eNOS uncoupling and increased production of reactive oxygen and nitrogen species (Zou et al., 2002; Bendall et al., 2014). In our present study, biopterin and BH2 levels were elevated in the female TZB group. In contrast, LV Me5THF levels were increased in the TZB-treated female group. Interestingly, Me5THF is a cofactor that prevents BH4 oxidation and eNOS uncoupling (Antoniades et al., 2006).

Notably, the LV levels of the L-arginine derivatives ADMA and SDMA were markedly higher in the TZB-treated females in our present study. ADMA is considered the strongest endogenous inhibitor of eNOS; therefore, it can limit NO bioavailability and increase the production of eNOS-derived reactive oxygen species (Oliva-Damaso et al., 2019; Liu et al., 2016). Additionally, SDMA has been reported to reduce NO production by decreasing intracellular L-arginine availability (Zou et al., 2002; Oliva-Damaso et al., 2019; Landmesser et al., 2003). SDMA is mainly excreted by the kidneys, and approximately 10%–20% of ADMA is eliminated via the kidneys; therefore, they are sensitive markers of renal function (Oliva-Damaso et al., 2019; Liu et al., 2016). In our present study, LV levels of uremic toxins, including ADMA, SDMA, and para-cresyl sulfate, were elevated in TZB-treated females and correlated with decreased creatinine clearance. Accordingly, several clinical studies have reported decreased renal function and focal segmental glomerulosclerosis in rare cases after TZB treatment, which may be more frequent in patients with preexisting cardiovascular disease or diabetes mellitus (Hakroush et al., 2021).

In the heart, NRG-1 binds more potently to the HER4 receptors, inducing HER2/HER4 heterodimer formation (Slavcheva and Angelov, 2023; Mohan et al., 2018). In our present study, in response to TZB, no significant changes in LV *Erbb2* expression at the transcript level were observed; however, ErbB2 expression was not measured at the protein level. Notably, mechanistic interpretations based solely on direct ErbB2 inhibition in rodents require careful consideration. Phillips et al. and Pegram and Ngo reported that TZB did not bind HER2 effectively in tissues from rats or mice (Le et al., 2022; Pegram and Ngo, 2006). In contrast, several studies in rodent models have reported the opposite (Riccio et al., 2009; Fedele et al., 2012; Ye et al., 2023; Sevieri et al., 2023; Pentassuglia et al., 2007; Singh et al., 2011; Zhu et al., 2025). Riccio et al. and Fedele et al. have demonstrated that Herceptin (i.e., trastuzumab) is capable of binding to ErbB2 receptor on rat H9c2 cardiomyoblasts, as well as rat and mouse ventricular cardiomyocytes, as shown by either immunoprecipitation or ELISA (Riccio et al., 2009; Fedele et al., 2012). Ye et al. have reported that TZB induced ferroptosis by suppressing the ErbB2/PI3K/AKT/Nrf2 signaling pathway and led to mitochondrial dysfunction via VDAC1 oligomerization in neonatal rat cardiomyocytes and female BALB/c mice (Ye et al., 2023). Moreover, Sevieri et al. have shown that ferritin nanoconjugates of TZB guided delivery across the blood-brain barrier, thereby promoting an antitumor response in brain metastasis originating from murine HER2-positive breast cancer (Sevieri et al., 2023). Despite the controversial results of small-animal studies on TZB’s ErbB2 binding, preclinical studies have reported cardiotoxic effects of TZB, suggesting additional direct interactions with CMs, immune-mediated mechanisms, and off-target effects (Kitani et al., 2019; Rodrigues et al., 2024; Bartolo et al., 2025; Mohan et al., 2018). Possible explanations include non-target-mediated toxicity related to the drug payload or Fc receptor-mediated clearance in specific tissues (Liu et al., 2026; Mohan et al., 2018); immune responses induced by TZB, such as antibody-dependent cellular cytotoxicity (ADCC) (Bartolo et al., 2025); and off-target structural remodeling or disruption of compensatory cellular pathways, potentially involving inflammation driven by reactive oxygen species, decreased antioxidant capacity, and altered cardiac metabolism (Eaton and Timm, 2023; Laird-Fick et al., 2020). In our present study, *Erbb4* and *Mtor* were overexpressed in the LV tissue of both TZB-treated groups, and *Nrg1* expression was significantly higher in TZB-treated females. Importantly, the NRG-1/HER4 pathway regulates the PI3K/AKT/mTOR signaling pathway, which is responsible for cell division and transcription, glucose and lipid metabolism, NO production, and mitochondrial respiration (Slavcheva and Angelov, 2023; Rochette et al., 2015; Zhu et al., 2025; Mohan et al., 2018).

Under physiological conditions, cardiac mitochondria are primarily fueled by fatty acids and, to a lesser extent, glucose; however, in HF, metabolic remodeling may occur (Necela et al., 2017). In our present study, the LV expression of genes crucial to glucose metabolism (i.e., *Slc2a4*, *Gapdh, Pkm, Ldha*) was decreased in both male and female TZB-treated rats. In alignment with our findings, other studies have also confirmed reduced glucose uptake and inhibitory effects on AMPK and *Ldha* in CMs after TZB treatment (Necela et al., 2017; Gordon et al., 2009; Ding et al., 2012). In the failing heart, metabolic remodeling can occur, with decreased glucose oxidation; meanwhile, fatty acid oxidation can be increased or decreased, depending on the etiology of HF (Sun et al., 2024). Fatty acid oxidation generally occurs in mitochondria and peroxisomes and provides most of the ATP required to meet the high-energy demand of CMs (Zheng et al., 2023). Carnitine palmitoyl-transferase 1A (*Cpt1a*) is located in the outer mitochondrial membrane, and it is the rate-limiting enzyme in fatty acid β-oxidation by regulating the transport of long-chain fatty acids (Zheng et al., 2023). In contrast, acyl-CoA oxidase 1 (*Acox1*) is the first, rate-limiting enzyme in the peroxisomal fatty acid oxidation (Wang-Heaton et al., 2024). In our present study, LV expression of *Cpt1a* and *Acox1* was decreased in response to TZB in both sexes. Although the current literature is contradictory regarding the effects of decreased *Cpt1* and *Acox1* expression in the heart (He et al., 2012; Mohan et al., 2017), inadequate fatty acid oxidation, accompanied by reduced glucose uptake and glycolysis, might lead to severe ATP depletion, further worsening TZB-induced cardiotoxicity.

Although the role of the kynurenine pathway, particularly kynurenic acid-related mechanisms, has already been investigated in the development of TZB resistance in HER2-positive breast cancer models, the literature is limited on the potential impact of TZB on Trp metabolism (Si et al., 2025). Trp is an essential amino acid that is processed through various metabolic pathways. The majority of Trp is broken down via the kynurenine pathway, resulting in several active metabolites, including NAD^+^ (Yang et al., 2024; Xie et al., 2020; Mangge et al., 2014; Peyraud et al., 2022). The kynurenine pathway has been widely explored in neurological disorders and cancer, but its role in the development of CVDs is still not fully understood (Yang et al., 2024; Mangge et al., 2014; Peyraud et al., 2022). In our present study, LV Trp levels were higher in the TZB-treated female animals. In alignment with our findings, several studies reported that females typically have higher circulating Trp levels; therefore, they have greater Trp availability to tissues, including the heart (Pais et al., 2023). Furthermore, in our present study, LV kynurenine levels were lower in the TZB-treated males, whereas kynurenic acid levels did not change after TZB treatment in either sex. However, there are still contradictions in the literature; elevated kynurenine levels are strongly associated with CVDs due to inflammatory and cardiac remodeling-promoting effects, while kynurenic acid has been linked to cardioprotective properties (Yang et al., 2024; Xie et al., 2020; Mangge et al., 2014; Lund et al., 2020). In our present study, LV levels of 3-hydroxykynurenine, quinolinic acid, and NAD^+^ were increased in the TZB-treated female group. Although quinolinic acid is mostly linked with increased nitro-oxidative stress, it is also a precursor for NAD^+^ formation (Yang et al., 2024; Xie et al., 2020; Mangge et al., 2014; Lund et al., 2020). NAD^+^ is needed for energy metabolism, redox homeostasis, DNA repair, stress resistance, and anti-inflammatory processes (Xie et al., 2020; Wu et al., 2024; Abdellatif et al., 2021). Recent studies have demonstrated reduced NAD^+^ levels in HF, a mechanism closely linked to metabolic remodeling and mitochondrial dysfunction (Xie et al., 2020). Notably, our present study quantified metabolite concentrations at a single terminal time point without assessing enzymatic activity or enzyme expression at the protein level. Metabolite abundance represents only the net balance of production, utilization, and clearance and therefore cannot be equated with pathway flux or adaptive responses.

In summary, in our present study, both sexes developed comparable TZB-induced chronic cardiotoxicity characterized by LV wall thinning, systolic and diastolic dysfunction, LV fibrosis, accompanied by the overexpression of several markers associated with nitro-oxidative stress and endothelial dysfunction (*e.g., Nos2, Nos3*), chronic inflammation *(e.g., Il1, Il6, Tgfb, Ctgf)*, as well as impaired glucose and fatty acid utilization *(i.e., repressed Slca2a1, Slc2a4, Gapdh, Pkm, Ldha, Cpt1a, Acox1)* at the transcript level. In the female TZB-treated group, increased LV concentrations of Trp metabolites associated with inflammation and nitro-oxidative stress (i.e., hydroxykynurenine and quinolinic acid) and NAD^+^ were observed. In contrast, in males, there was no significant difference in Trp and most of its measured metabolite levels (i.e., kynurenic acid, anthranilic acid, 3-hydroxykynurenine, quinolinic acid, and NAD^+^). However, kynurenine and xanthurenic acid levels were lower in the male TZB-treated group. In conclusion, our present chronic cardiotoxicity model appears valuable for exploring cardiotoxic phenotypes associated with TZB treatment. However, LV Trp metabolite concentrations showed sex-divergent alterations; the functional significance and potential adaptive roles of these molecular differences remain to be elucidated in future longitudinal and mechanistic studies involving multiple time points and functional measurements. We believe that our present study still provides valuable insights into TZB-induced chronic cardiotoxicity and may help initiate further discussion on the development and application of novel pharmacological agents to attenuate TZB-associated cardiac side effects.

Our present study is not without limitations. The pathomechanism of TZB-induced chronic cardiotoxicity remains incompletely understood and is still under active investigation. Further studies using complementary models with validated HER2 binding will be necessary to clarify the precise molecular mechanisms underlying TZB-induced chronic cardiotoxicity. The aim of our present study was to determine whether differences exist in LV concentrations of selected Trp metabolites, rather than to demonstrate the mechanisms underlying these differences. *In vivo* administration of synthetic inhibitors or analogs of Trp metabolites to TZB-treated and control animals could clarify the mechanistic role of altered Trp metabolism and its potential direct links to oxidative stress, inflammation, and sex-based differences in future studies of TZB-induced chronic cardiotoxicity. The potential protective role of female hormones, particularly estrogen, in TZB-induced cardiotoxicity was not thoroughly explored in this study (e.g., by accounting for the estrus cycle, ovariectomy, or estrogen administration in males). While previous studies suggest that estrogen may offer cardioprotective effects in HF, our focus was more on the descriptive characterization of sex-based differences in response to TZB. A detailed analysis of hormonal levels could provide further insight into the sex-based differences in the development of TZB-induced chronic cardiotoxicity. The translational applicability of our findings is limited by the absence of aging and cardiovascular risk factors, as well as by the lack of cancer. Moreover, our present findings are limited to the follow-up time points (i.e., 12 and 19 weeks for echocardiography, and 20 weeks for histology and biochemical measurements in tissue samples) and do not rule out the possibility of a more pronounced difference between the two sexes at a later time point. Importantly, our exploratory study is mainly descriptive and correlative; therefore, the observed metabolic alterations, particularly changes in Trp metabolite levels, cannot be interpreted as direct causes or consequences of TZB-induced chronic cardiotoxicity. Therefore, a more detailed exploration of the molecular mechanisms, including analysis of gene expression at the transcript and protein levels, post-transcriptional modifications of proteins, measurements of enzymatic activity or metabolite flux underlying the observed sex-based differences in Trp metabolism, would be essential to elucidate the pathways and interactions between different cell types involved in TZB-induced chronic cardiotoxicity in future studies.

## Data Availability

The raw data supporting the conclusions of this article will be made available by the authors, without undue reservation.
