# Correction: Investigation of potential sex-based differences in trastuzumab-induced chronic cardiotoxicity in a rat model

**DOI:** 10.3389/fphar.2026.1926577

**Published:** 2026-08-14

**Authors:** Réka Losonczi, Zsolt Galla, Merse Kis, Klaudia Kupecz, Dávid Volford, Andrea Siska, Réka Somogyi, Imre Földesi, Gábor Cserni, András Kriston, Ferenc Kovács, Péter Horváth, Péter Monostori, Zsuzsanna Kahán, Márta Sárközy

**Affiliations:** 1 Cardiovascular Research Group, Department of Pathophysiology, Albert Szent-Györgyi Medical School, University of Szeged, Szeged, Hungary; 2 Department of Biochemistry, Albert Szent-Györgyi Medical School, University of Szeged, Szeged, Hungary; 3 Metabolic and Newborn Screening Laboratory, Department of Pediatrics, Albert Szent-Györgyi Medical School, University of Szeged, Szeged, Hungary; 4 Department of Laboratory Medicine, Albert Szent-Györgyi Medical School, University of Szeged, Szeged, Hungary; 5 Department of Pathology, Albert Szent-Györgyi Medical School, University of Szeged, Szeged, Hungary; 6 Synthetic and Systems Biology Unit, HUN-REN Biological Research Centre, Szeged, Hungary; 7 Single-Cell Technologies Ltd., Szeged, Hungary; 8 Institute for Molecular Medicine Finland, University of Helsinki, Helsinki, Finland; 9 Institute of AI for Health, Helmholtz Zentrum München, Neuherberg, Germany; 10 Department of Oncotherapy, Albert Szent-Györgyi Medical School, University of Szeged, Szeged, Hungary

**Keywords:** glucose and fatty acid metabolism, heart failure, kynurenine pathway, trastuzumab, trastuzumab-induced chronic cardiotoxicity, tryptophan and arginine metabolites

Author Péter Monostori was erroneously assigned to affiliation 4. Department of Laboratory Medicine, Albert Szent-Györgyi Medical School, University of Szeged, Szeged, Hungary. The correct affiliation for this author is 3. Metabolic and Newborn Screening Laboratory, Department of Pediatrics, Albert Szent-Györgyi Medical School, University of Szeged, Szeged, Hungary.

The original version of this article has been updated.

