# Supplementary material for: Investigation of potential sex-based differences in trastuzumab-induced chronic cardiotoxicity in a rat model
Source: Front Pharmacol. 2026 Jun 26;17:1809964. doi: 10.3389/fphar.2026.1809964 (PMC13349826; doi:10.3389/fphar.2026.1809964)
Supplement: Supplementary file 2 [file Supplementaryfile1.docx]

Supplementary Material

**Supplementary Figure 1** Left ventricular morphologic alterations in response to trastuzumab (TZB) treatment assessed by echocardiography in both sexes at week 13. (A) Representative M-mode images, (B) septal wall thickness in diastole (SWTd), (C) septal wall thickness in systole (SWTs), (D) posterior wall thickness in diastole (PWTd), (E) posterior wall thickness in systole (PWTs), (F) left ventricular end-diastolic diameter (LVEDD), and (G) left ventricular end-systolic diameter (LVESD). Values are presented as mean ± S.E.M., *p < 0.05, **p < 0.01, ***p < 0.001, ****p < 0.0001, n = 6-7, Two-Way ANOVA, Holm-Sidak post hoc test. CT: Control and TZB: trastuzumab. Each circle corresponds to one individual data point.

**Supplementary Figure 2** Left ventricular functional alterations in response to trastuzumab (TZB) treatment assessed by echocardiography in both sexes at week 13. (A) left ventricular end-diastolic volume (LVEDV), (B) left ventricular end-systolic volume (LVESV), (C) stroke volume (SV), (D) ejection fraction (EF), (E) heart rate (HR), (F) cardiac output (CO), (G) representative Doppler and tissue Doppler images, (H) peak early diastolic mitral inflow velocity (E), (I) early diastolic mitral annular velocity (e'), and (J) E/e'. Values are presented as mean ± S.E.M., *p < 0.05, **p < 0.01, ***p < 0.001, ****p < 0.0001, n = 6-7, Two-Way ANOVA, Holm-Sidak post hoc test. CT: Control and TZB: trastuzumab. Each circle corresponds to one individual data point.

**Supplementary Figure 3** Alterations in the LV concentrations of cofactors related to endothelial nitric oxide synthase (eNOS) in response to TZB treatment in both sexes. (A) Biopterin (BIO), dihydroxybiopterin (BH2), and 5’-methyltetrahydrofolic acid (Me5THF). Values are presented as mean ± SEM, *p < 0.05, **p < 0.01, ***p < 0.001, ****p < 0.0001, n= 5-7, Two-Way ANOVA, Holm-Sidak *post hoc* test. CT: Control and TZB: trastuzumab. Each circle corresponds to one individual data point.

**Supplementary Figure 4** Changes in the LV expression of the *Nrg1, Erbb2, Erbb4*, and *Mtor* in response to TZB treatment in both sexes. (A) neuregulin 1 (*Nrg1*), (B) human epidermal growth factor receptor 2 (*ErbB2*), (C) human epidermal growth factor receptor 4 (*ErbB4*), and (D) mammalian target of rapamycin (*Mtor*). Values are presented as mean ± SEM, *p < 0.05, **p < 0.01, n = 5-7, Two-Way ANOVA, Holm-Sidak *post hoc* test. CT: Control and TZB: trastuzumab. Each circle corresponds to one individual data point.

**Supplementary Figure 5** Alterations in LV Trp metabolite concentrations in the serotonine and indole pathways in response to TZB treatment in both sexes**.** (A) Serotonin, (B) indol-3-acetic acid, (C) indol-3-lactic acid, (D) indol-3-propionic acid, (E) 5-OH indol acetic acid, and (F) indol-3-carboxaldehyde. Values are presented as mean ± SEM, *p < 0.05, ***p < 0.001, n = 5-7, Two-Way ANOVA, Holm-Sidak *post hoc* test. CT: Control and TZB: trastuzumab. Each circle corresponds to one individual data point.
